# The Relationship between the Hounsfield Units Value of the Upper Instrumented Vertebra and the Severity of Proximal Junctional Fracture after Adult Spinal Deformity Surgery

**DOI:** 10.3390/medicina59061086

**Published:** 2023-06-04

**Authors:** Norichika Yoshie, Keishi Maruo, Fumihiro Arizumi, Kazuya Kishima, Tomoyuki Kusukawa, Toshiya Tachibana

**Affiliations:** Department of Orthopaedic Surgery, Hyogo Medical University, 1-1 Mukogawa-cho, Nishinomiya 663-8501, Japan

**Keywords:** adult spinal deformity, bone mineral density, fracture, Hounsfield units, proximal junctional kyphosis, upper instrumented vertebra

## Abstract

*Background and Objectives*: In this retrospective cohort study, we investigate associations between the Hounsfield units (HU) value of upper instrumented vertebra (UIV) and proximal junctional kyphosis (PJK) after adult spinal deformity (ASD) surgery. *Materials and Methods*: The cohort consisted of 60 patients (mean age 71.7 years) who underwent long instrumented fusion surgery (≥6 vertebrae) for ASD with at least 1 year of follow-up. The preoperative bone mineral density (BMD) measured on DXA scans, the HU values at UIV and UIV+1, and the radiographic parameters were compared between the PJK and non-PJK groups. The severity of UIV fracture was assessed using a semiquantitative (SQ) grade. *Results*: PJK occurred in 43% of patients. No significant differences in patient age, sex, BMD, and preoperative radiographic parameters were observed between the PJK and non-PJK groups. The HU values of the UIV (103.4 vs. 149.0, *p* < 0.001) and UIV+1 (102.0 vs. 145.7, *p* < 0.001) were significantly lower in the PJK group. The cutoff values of HU at UIV and UIV+1 were 122.8 and 114.9, respectively. Lower HU values at UIV (Grade 1: 134.2, Grade 2: 109.6, Grade 3: 81.1, *p* < 0.001) and UIV+1 (Grade 1: 131.5, Grade 2: 107.1, Grade 3: 82.1, *p* < 0.001) were associated with severe SQ grade. *Conclusions*: Lower HU values at UIV and UIV+1 had a negative impact on signal incidence of PJK and were correlated with the severity of UIV fractures. Preoperative treatment of osteoporosis seems necessary for preoperative UIV HU values less than 120.

## 1. Introduction

As the aging population grows, the number of elderly patients with adult spinal deformities (ASDs) is increasing accordingly. Long instrumented fusion surgery for ASD patients with severe disability has become the gold standard in the past few decades. However, proximal junctional kyphosis (PJK) remains a major and severe complication after ASD surgery [1]. As no effective method for preventing PJK has yet been established, it is expected that the number of PJK cases continues to increase, too. The incidence of PJK in adults has been reported with a range of rates from 9% to 43% [1,2,3,4,5,6,7,8,9,10,11,12,13,14,15,16]. Several studies have identified multiple factors associated with PJK, including patient-, surgery-, and radiographic-related factors [3,6,7,8,10,15,16,17]. In particular, osteoporosis is a major cause of PJK after ASD surgery [6,15]. Hence, it is crucial to evaluate osteoporosis preoperatively. Dual-energy X-ray absorptiometry (DXA) is typically used to evaluate bone mineral density (BMD). However, DXA was reported to encounter some limitations, as it cannot exactly represent the bone quality of the lumbar spine because degenerative changes in the vertebrae or the calcification of soft tissues or adjacent vascular structures may lead to overestimating its real value [18,19,20]. To avoid this issue, a simple method for measuring computed tomography attenuation in Hounsfield units (HU) has been proposed in the recent literature for bone quality assessment [21,22,23,24,25,26,27]. HU is only measured in trabecular bones of the vertebral body, avoiding the degenerative changes recorded by standard computed tomography scans (CT scans) [21]. Several studies demonstrated that the PJK group after ASD surgery had lower HU values of upper instrumented vertebra (UIV) than the non-PJK group [28,29]; however, they only took into consideration HU values measured on preoperative CT because DXA is not routinely performed as a preoperative screening for osteoporosis. This situation has motivated us to routinely perform preoperative DXA scans to detect osteoporosis before ASD surgery in the framework of this study, aiming to uncover associations between osteoporosis assessed with HU values on preoperative CT and DXA scans and PJK and to reveal yet unknown relationships between the HU values and the severity of corresponding UIV fractures. The aim of this study is to investigate whether lower HU values at UIV and UIV+1 are reliable indicators for the incidence of PJK and whether they are correlated with the severity of UIV fractures.

## 2. Materials and Methods

### 2.1. Patient Population

We retrospectively reviewed 60 consecutive patients who underwent long instrumented fusion surgery from the thorax to the pelvis between 2014 and 2018 at our institute. The patient group consisted of 15 men and 45 women, a mean age at surgery of 71.7 ± 7.3 years (range 41 to 85 years) and had a mean follow-up of 37.1 ± 14.5 months. Inclusion criteria were patients with spinal deformity older than 40 years, minimum six vertebrae fused—with fusion to pelvis, availability of a preoperative CT scan within 3 months of the index surgery, minimum 1 year of clinical follow-up, and complete radiographic follow-up. The preoperative diagnoses were degenerative lumbar kyphoscoliosis in 37 subjects, kyphotic deformity after vertebral fracture in 9 cases, iatrogenic flat back syndrome in 12 patients, and Parkinson’s disease in the remaining 2. This study included patients who had both primary and revision surgery, and subjects with incomplete radiographic follow-up were excluded. This study was approved by the IRB (Institutional Review Board) of the authors’ affiliated institution. Informed consent was obtained from all participating patients. Patient demographics and surgical data were obtained from medical records and operative reports.

### 2.2. Surgical Procedure for ASD

The employed surgical procedure was decided based on the rigidity of the spine. Most patients underwent two-stage surgery. Firstly, lateral lumbar interbody fusion (LIF) surgery was performed using oblique lumbar interbody fusion or extreme lateral interbody fusion (XLIF). One week after LIF, posterior correction and fusion surgery were performed with standard open approach using facetectomy at lower lumbar spine. Then, transforaminal lumbar interbody fusion was performed at L5–S1. Finally, the posterior correction was performed using the cantilever technique. Three column osteotomy surgery was indicated in patients with iatrogenic flat back syndrome, which was performed in the posterior surgery. Combined anterior–posterior surgery was performed in 42 (70%) patients. Pedicle subtraction osteotomy was performed in 10 (17%) patients and vertebral column resection in 4 patients to achieve deformity correction. All patients were undergoing iliac fixation using S-2 alar-iliac (S2AI) screws or traditional iliac screws. Pelvic fixation with S2AI screws was used in 39 (65%) patients, and traditional iliac screws were used in 21 patients (35%). The UIV instrumentation involved hooks in 39 (65%) patients and pedicle screws in 21 (35%) patients. A 5.5 mm titanium rod was used in 40 (67%) patients, and a 5.5 mm cobalt-chrome rod was used in 20 (23%) patients.

### 2.3. Radiographic Measurements and Data Collection

All patients had full-length free-standing radiographs, DXA, and standard CT scans 3 months before surgery. We performed full-length free-standing radiographs preoperatively and within 1 year postoperatively. Radiographic parameters included thoracic kyphosis (TK, T5–T12), lumbar lordosis (LL, L1–S1), pelvic incidence (PI), pelvic tilt (PT), sagittal vertical axis (SVA), and proximal junctional angle (PJA), which is defined as the caudal endplate of the UIV to the cephalad endplate of 2 supraadjacent vertebrae above the UIV (UIV+2). PJK was defined as a sagittal Cobb angle between the UIV and the UIV+2 of 10° or greater and at least 10° greater than the pre-operative measurement [2]. DXA scans were performed in each patient’s lumbar spine and proximal femur. The HU value was evaluated at UIV and UIV+1 in the vertebral body using preoperative standard CT scans [21]. All measurements were performed using a helical 128-channel CT scanner (SOMATOM Definition Edge; Siemens Healthineers, Germany, tube voltage 120 kV, slice thickness of 1 mm with a 0.7 mm interval).

We used a standard picture archiving and communication system software (FUJIFILM Corporation, Japan), and an elliptical region of interest (ROI) was drawn on an axial slice in an oval pattern only on the cancellous bone, excluding cortical bones.

**Figure 1:**
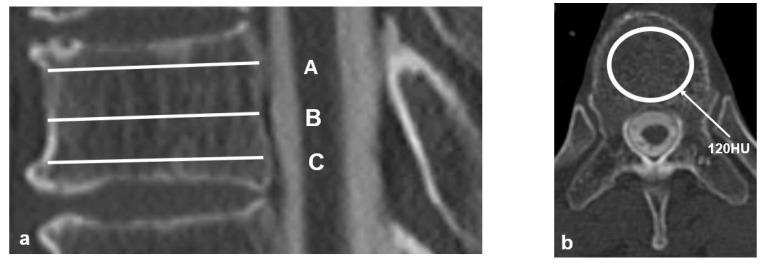
Technique for obtaining Hounsfield units (HU) values. (**a**) Three HU measurements were evaluated as follows: (A) immediately inferior to the superior end plate; (B) middle of the vertebral body; and (C) immediately superior to the inferior end plate. (**b**) The oval pattern indicates an elliptical region of interest (ROI), which evaluates HU values on an axial image avoiding cortical bone.

The HU values were evaluated at three points for each vertebral body as follows: immediately inferior to the superior endplate (Figure 1; line A), middle of the vertebral body (Figure 1; line B), and immediately superior to the inferior endplate (Figure 1; line C). These three values were then averaged for each individual vertebral body (Figure 1). The UIV fractures were evaluated on the basis of a semiquantitative (SQ) technique, which used a standard CT scan, and was graded on visual as follows: normal (grade 0), mildly deformed (grade 1, approximately 20–25% reduction in anterior, middle, and/or posterior height and a reduction in area 10–20%), moderately deformed (grade 2, approximately 25–40% reduction in any height and a reduction in area 20–40%), and severely deformed (grade 3, approximately 40% reduction in any height and area) [30]. We examined age, sex, teriparatide usage, BMD measured on DXA scans, HU, and SQ grade as patients’ factors and UIV region (UT: upper thoracic, T1–T6, LT: lower thoracic, T7–T12), iliac fixation, and UIV fixation as surgery-related factors. Moreover, we examined TK, LL, PI, PT, SVA, and PJA as radiographic parameters.

### 2.4. Statistical Analysis

Statistical analysis was performed using JMP**^®^** 14 (SAS Institute Inc., Cary, NC, USA). The Student t test was used to assess the significance of differences for continuous measures, and an χ^2^ test was used for dichotomous variables. The significance level was set at 5%. Receiver operating characteristic (ROC) curve analysis and area under the curve (AUC) were used to evaluate the cutoff values of HU values in PJK.

## 3. Results

**Figure 2:**
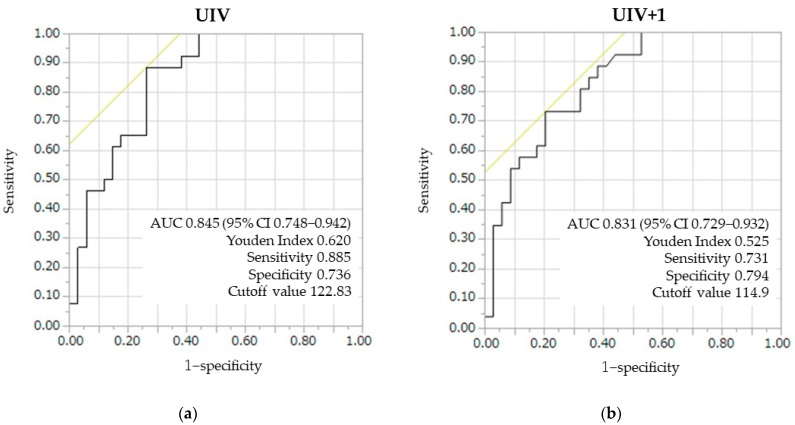
Receiver operating characteristic curves for HU values of (**a**) UIV and (**b**) UIV+1. Yellow line: 45−degree line tangent to ROC curve.

**Figure 3:**
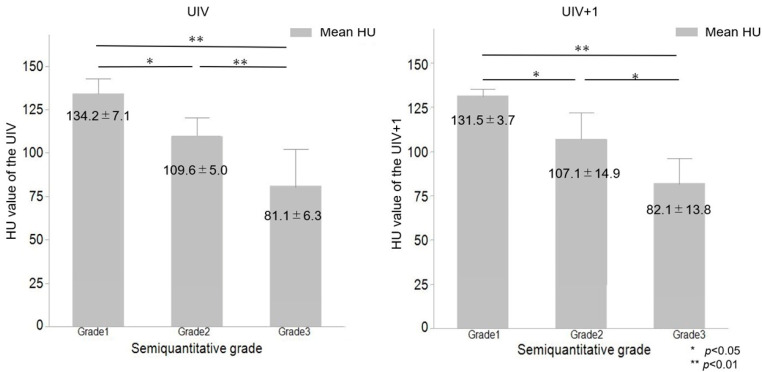
Association between HU value of UIV and severity of SQ grades.

PJK occurred in 26 of the 60 (43%) patients. Fracture at the UIV occurred in 11 patients, fracture at UIV+1 and UIV-1 occurred in 6 and 3 patients, respectively, and instrumentation problems occurred in 8 patients. Instrumentation problems occurred in eight patients, including hook dislodgement in four patients, screw backout or cutout in four patients, and with rod dislodgement in two patients. Four patients with instrumentation problems had UIV or UIV+1 fractures. Only instrumentation problems occurred in four (15%) patients. Revision surgery for PJK, with proximal extension of the instrumentation, was required in five (8%) patients. The average time to revision surgery was 12.2 ± 2.6 months. Among the five patients undergoing revision surgery, three had back pain and two had a sudden onset of paralysis (Frankel grade C) with UIV and UIV+1 fracture. There were no significant differences in patient age (73.2 ± 4.8 years vs. 70.5 ± 8.7 years, *p* = 0.156) and sex (female 85% vs. 68%, *p* = 0.228) between the PJK and non-PJK group as well as no significant differences in the UIV region (LT: 92% vs. 88%, *p* = 0.689), type of pelvic fixation (S2AI screws: 65% vs. 65%, *p* = 0.956), or UIV fixation (hook: 72% vs. 60%, *p* = 0.416). Thirty-five patients (60%) received preoperative drug treatment for osteoporosis, twenty-nine (48%) of them teriparatide, six (10%) received bisphosphonate, and one denosumab. Patients with a preoperative BMD less than 70% of the young adult mean (YAM) received teriparatide. There was no significant difference in the use of teriparatide between the PJK and non-PJK group (65% vs. 38%, *p* = 0.067) (Table 1 and Table 2). There were no significant differences in the preoperative and postoperative (1 year) radiographic parameters between the PJK group and non-PJK group, except for postoperative PJA (Table 3). The HU values at UIV were significantly lower in the PJK than those in the non-PJK group (103.4 ± 22.9 vs. 149.0 ± 42.6, *p* < 0.001), and so were the HU values at UIV+1 (102.0 ± 22.8 vs. 145.7 ± 43.0, *p* < 0.001); however, we did not notice any significant difference in BMD of the Lumbar spine and proximal femur between the PJK and non-PJK groups (Lumbar spine: 0.874 ± 0.130 vs. 0.900 ± 0.250, *p* = 0.704, Proximal femur: 0.661 ± 0.110 vs. 0.720 ± 0.230, *p* = 0.350) (Table 4). ROC curve analysis demonstrated that the AUC for HU at UIV was 0.845. The cutoff value was 122.8 (sensitivity: 0.885, specificity: 0.736), according to the Youden Index. The AUC for HU at UIV+1 was 0.831. The cutoff value was 114.9 (sensitivity: 0.731, specificity: 0.794), according to the Youden Index (Figure 2). Evaluated according to the severity of SQ grade in patients with PJK, the HU values at UIV (134.2 ± 7.1, 109.6 ± 5.0, 81.1 ± 6.3, *p* < 0.001) and UIV+1 (Grade 1: 131.5 ± 3.7, Grade 2: 107.1 ± 14.9, Grade 3: 82.1 ± 13.8, *p* < 0.001) significantly decreased with the increasing severity of the SQ grade (Figure 2 and Figure 3).

## 4. Discussion

In this study, patients with PJK after ASD surgery had significantly lower HU values at UIV or UIV+1 than the ones from the non-PJK group, even though no significant differences in BMD were noticed. Previously, it was demonstrated that HU may be useful for risk assessment of revision surgery on ASD, as it was significantly lower at T8 and T9 in elderly patients who required revision surgery [31]. In particular, the values of HU at UIV and UIV+1 on preoperative CT were effective for predicting PJK in ASD surgery [28,29]. Moreover, HU values at UIV and UIV+1 lower than 120 have shown a significant risk for postoperative bony PJK [28]. Another study concluded that the optimal HU values by the Youden Index were 104 HU at UIV, 113 HU at UIV+1, and 110 HU at UIV+2, with these values being useful adjuncts when planning surgical correction of ASD on preoperative CT [29]. Hiyama reported evaluating global alignment and proportion score (GAP score) involving age. The study clarified the relationships between proximal junctional failure and HU values in elderly patients [32]. However, different to our investigation, these two studies did not evaluate preoperative DXA scans. For comparison, in our work, the cutoff values of HU at UIV and UIV+1 were 122.8 and 114.9, respectively. We routinely assessed preoperative BMD because the average age of our patients (71.7 years) was approximately 10 years higher than in the two previous studies. In addition, a total of 48% of patients received preoperative teriparatide therapy when their BMD was less than 70% of the YAM. However, there were no significant differences in the use of teriparatide between the PJK and non-PJK groups, and it seems that DXA may have underestimated the magnitude of vertebral bone quality, leading to false-negative rates of osteoporosis. Therefore, preoperative assessment of HU values on both CT and DXA scans may be necessary to evaluate osteoporosis in elderly patients with ASD surgery. 

Various risk factors, such as patient-, surgery-related, and radiographic factors, were found to be possible causes of developing PJK. In our study, patient factors, including age, sex, and preoperative and postoperative radiographical factors, did not reveal any correlation with PJK, with low HU values at UIV or UIV+1 being the only predictor associated with PJK. On the other hand, in the recent literature focused on osteoporosis, in particular, on low BMD, as a major risk factor for PJK [15]. Moreover, old age and osteopenia/osteoporosis were revealed as significant independent risk factors for PJK [15,17,27]. Additionally, vertebral HU has been shown to detect osteoporosis. The L1 vertebral body was mostly chosen for HU measurements, with a threshold value of 110 HU [27]. Patients with acute vertebral fragility fractures had significantly lower HU values (average 66.0) than those without fractures [33]. Accordingly, our study showed that a severe SQ grade led to a significantly lower HU value at UIV (Grade 2; average 109.6 HU and Grade 3; average 81.1 HU) or at UIV+1 (Grade 2; average 107.1 HU and Grade 3; average 82.1 HU). To the best of our knowledge, no previous work examined possible connections between HU values and the severity of fractures at UIV or UIV+1, with only a significant negative correlation between the HU value and PJK angle being known [28]. Our findings suggest that low HU values could signal the possibility of severe PJK after ASD surgery; however, more investigations are necessary for a better understanding of this relationship.

There is a lack of robust clinical evidence on how to prevent PJK after ASD surgery. Measures such as posterior polyethylene tethers [34], vertebroplasty at UIV [35], UIV hook fixation and terminal rod contouring [36], and teriparatide treatment [37] were employed for preventing PJK; however, only limited success has been reported so far—with only the latter method leading to significantly increased BMD at UIV+1, whereas the teriparatide treatment group had a significantly lower rate of PJK. On the other hand, our study results suggest that teriparatide treatment did not significantly reduce PJK. Several possible reasons for this result could be other factors related to PJK, different timing of teriparatide treatment, or inadequate evaluation of osteoporosis using BMD because if the patients had a BMD more than 70% of the YAM and an HU value at UIV or UIV+1 less than 110, we recommended postponing surgery and preoperative osteoporosis treatment with osteoanabolic agents, such as teriparatide, abaloparatide, and romosozumab for more than 3 months before ASD surgery.

This study has some limitations. First, we mention a small number of patients with ASD from a single center considered in our retrospective investigation and it is therefore difficult to calculate propensity-score matching. A larger sample size from multiple centers used in a prospective study would be necessary for a more accurate analysis of the relationship between HU values and PJK. Second, our study ignored the duration of the teriparatide treatment, as the majority of patients started it when surgery had already been planned. The duration of the treatment needs to be taken into consideration to evaluate the effect of teriparatide on the considered patients. Third, we did not calculate intraobserver and interobserver variability in HU measurement, whereas Schreiber et al. demonstrated intraobserver and interobserver reliability with interclass correlation coefficients of 0.964 and 0.975, respectively [21]. When comparing sizes of ROIs, Romme et al. demonstrated an excellent agreement in HU values between ROIs of sizes 50, 100, and 200 mm2 with interclass coefficient > 0.99 in each comparison [38]. Fourth, the ratio of PJK was relatively high, whereas no standardized definition exists for PJK. Ton analyzed nine definitions representative of the most commonly cited definitions and their variations. The incidence of PJK significantly decreased with stricter definitions and they reported that the ratio of PJK using the definition established by Glattes was 40% [39]. Furthermore, our study involved an elderly population with an average age of 71.7 years old and the preoperative diagnosis included nine cases of kyphotic deformity after vertebral fracture and two cases of Parkinson’s disease. Fifth, our study included various types of surgery, such as three column osteotomy or LIF and posterior spinal correction surgery, without treating the mechanical stress at UIV in a different manner, with respect to distinct surgical procedures. In addition, rod diameter, rod material, type of UIV fixation, and type of pelvic fixation were chosen by the surgeons’ preference. There was a tendency for more hooks in the failure group. Little is known regarding whether hooks or screws have an advantage on the UIV in ASD surgery. We used hooks with posterior polyethylene tethers until 2016. Nonetheless, there was no clear effect to prevent PJK, and we changed to using screws with posterior polyethylene tethers in 2017. However, there were no significant differences in patient-, surgery-related, and radiographic factors. Our investigation shows that lower HU values at UIV and UIV+1 are reliable indicators for the incidence of PJK and that they are correlated with the severity of UIV fractures.

## 5. Conclusions

Our study revealed that while there were no significant differences in the BMD between the PJK and non-PJK groups, the HU values at UIV and UIV+1 were significantly lower in the PJK group than those in the non-PJK group. Moreover, patients with a severe SQ grade of a UIV fracture showed lower HU values as well. Therefore, one can safely conclude that the HU values at UIV and UIV+1 are useful predictors of PJK after long instrumented spinal fusion surgery for ASD, and preoperative treatment of osteoporosis seems necessary for UIV preoperative HU values less than 120.

## Figures and Tables

**Table 1:** Baseline characteristics in the PJK group (*n* = 26) and non-PJK group (*n* = 34).

Characteristic	PJK (*n* = 26)	Non-PJK (*n* = 34)	*p* Value
Age, mean ± SD (years)	73.2 ± 4.8	70.5 ± 8.7	0.156
Sex (*n*, % female)	22 (85%)	23 (68%)	0.228
UIV			0.689
UT: T1–6 (*n*, %)	2 (8%)	4 (12%)	
LT: T7–12 (*n*, %)	24 (92%)	30 (88%)	
Iliac fixation			0.956
Iliac screw (*n*, %)	9 (35%)	12 (35%)	
S2AI screw (*n*, %)	17 (65%)	22 (65%)	
UIV fixation			0.416
Hook (*n*, %)	18 (72%)	21 (60%)	
Screw (*n*, %)	7 (28%)	14 (40%)	
Three column osteotomy (*n*, %)	4 (29%)	8 (71%)	0.235
Teriparatide usage	65%	38%	0.067

UT, upper thoracic; LT, lower thoracic; PJK, proximal junctional kyphosis; UIV, upper instrumented vertebra; SD, Standard deviation.

**Table 2:** Mechanisms of PJK.

Mechanisms of PJK	*n* (Total = 26)
UIV+1 fracture (*n*, %)	6 (23%)
UIV fracture (*n*, %)	11 (42%)
UIV-1 fracture (*n*, %)	3 (12%)
Instrumentation problem (*n*, %)	8 (31%)

PJK, proximal junctional kyphosis; UIV, upper instrumented vertebra.

**Table 3:** Comparison of preoperative and postoperative (1 week and 1 year) radiographic parameters in two groups.

Characteristic	PJK (*n* = 26)	Non-PJK (*n* = 34)	*p* Value	Characteristic	PJK (*n* = 26)	Non-PJK (*n* = 34)	*p* Value
TK				SVA			
Preoperative, mean ± SD (°)	14.7 ± 15.9	18.4 ± 10.6	0.278	Preoperative, mean ± SD (mm)	133.4 ± 54.3	139.9 ± 60.1	0.679
Postoperative 1 week, mean ± SD (°)	36.4 ± 11.3	36.8 ± 10.1	0.883	Postoperative 1 week, mean ± SD (mm)	23.1 ± 35.5	33.5 ± 28.3	0.291
Postoperative 1 year, mean ± SD (°)	35.9 ± 9.3	34.6 ± 9.1	0.591	Postoperative 1 year, mean ± SD (mm)	24.9 ± 7.4	38.5 ± 6.5	0.172
LL				PI–LL			
Preoperative, mean ± SD (°)	6.7 ± 15.2	6.3 ± 13.9	0.900	Preoperative, mean ± SD (°)	41.8 ± 15.5	41.4 ± 13.9	0.918
Postoperative 1 week, mean ± SD (°)	48.9 ± 7.8	47.0 ± 11.6	0.471	Postoperative 1 week, mean ± SD (°)	−0.4 ± 9.4	0.7 ± 10.1	0.680
Postoperative 1 year, mean ± SD (°)	47.1 ± 7.3	45.8 ± 10.3	0.571	Postoperative 1 year, mean ± SD (°)	1.4 ± 8.2	1.9 ± 7.1	0.803
PT				PJA			
Preoperative, mean ± SD (°)	34.0 ± 9.7	33.6 ± 8.6	0.885	Preoperative, mean ± SD (°)	6.6 ± 6.2	6.5 ± 4.2	0.478
Postoperative 1 week, mean ± SD (°)	22.2 ± 8.1	19.3 ± 7.0	0.138	Postoperative 1 week, mean ± SD (°)	8.3 ± 6.0	7.9 ± 4.0	0.784
Postoperative 1 year, mean ± SD (°)	20. 6± 6.1	18.5 ± 4.2	0.127	Postoperative 1 year, mean ± SD (°)	24.1 ± 9.1	10.2 ± 24.1	<0.001 *
SS				PI			
Preoperative, mean ± SD (°)	14.5 ± 11.0	14.0 ± 8.8	0.843	Preoperative, mean ± SD (°)	48.5 ± 6.7	47.7 ± 7.3	0.646
Postoperative 1 week, mean ± SD (°)	26.3 ± 7.8	29.9 ± 13.8	0.236				
Postoperative 1 year, mean ± SD (°)	27.9 ± 7.2	29.1 ± 6.7	0.500				

PJK, proximal junctional kyphosis; TK, thoracic kyphosis (T5–T12); LL, lumbar lordosis (L1–S1); PI, pelvic incidence; PT, pelvic tilt; SVA, sagittal vertical axis; PJA proximal junctional angle; SD, Standard deviation; * *p* < 0.01.

**Table 4:** Comparison of BMD, HU value of UIV and UIV+1 between two groups.

Characteristic	PJK (*n* = 26)	Non-PJK (*n* = 34)	*p* Value
BMD of the Lumbar spine, mean ± SD (g/cm^2^)	0.874 ± 0.130	0.900 ± 0.250	0.704
BMD of the Proximal femur, mean ± SD (g/cm^2^)	0.661 ± 0.110	0.720 ± 0.217	0.350
HU value of the UIV, mean ± SD	103.4 ± 22.9	149.0 ± 42.6	<0.001 *
HU value of the UIV+1, mean ± SD	102.0 ± 23.8	145.7 ± 43.0	<0.001 *

PJK, proximal junctional kyphosis; HU, Hounsfield units; UIV, upper instrumented vertebra; BMD, bone mineral density; SD, Standard deviation; * *p* < 0.01.

## Data Availability

The data presented in this study are available on request from the corresponding author. The data are not publicly available due to privacy and ethical restrictions.

## References

[1] Hyun S.J., Lee B.H., Park J.H., Kim K.J., Jahng T.A., Kim H.J. (2017). Proximal junctional kyphosis and proximal junctional failure following adult spinal deformity surgery. Korean J. Spine.

[2] Glattes R.C., Bridwell K.H., Lenke L.G., Kim Y.J., Rinella A., Edwards C. (2005). Proximal junctional kyphosis in adult spinal deformity following long instrumented posterior spinal fusion: Incidence, outcomes, and risk factor analysis. Spine.

[3] Kim Y.J., Bridwell K.H., Lenke L.G., Glattes C.R., Rhim S., Cheh G. (2008). Proximal junctional kyphosis in adult spinal deformity after segmental posterior spinal instrumentation and fusion: Minimum five-year follow-up. Spine.

[4] Denis F., Sun E.C., Winter R.B. (2009). Incidence and risk factors for proximal and distal junctional kyphosis following surgical treatment for Scheuermann kyphosis: Minimum five-year follow-up. Spine.

[5] Pichelmann M.A., Lenke L.G., Bridwell K.H., Good C.R., O’Leary P.T., Sides B.A. (2010). Revision rates following primary adult spinal deformity surgery: Six hundred forty-three consecutive patients followed-up to twenty-two years postoperative. Spine.

[6] Yagi M., Akilah K.B., Boachie-Adjei O. (2011). Incidence, risk factors and classification of proximal junctional kyphosis: Surgical outcomes review of adult idiopathic scoliosis. Spine.

[7] Yagi M., King A.B., Boachie-Adjei O. (2012). Incidence, risk factors, and natural course of proximal junctional kyphosis: Surgical outcomes review of adult idiopathic scoliosis. Minimum 5 years of follow-up. Spine.

[8] Kim H.J., Lenke L.G., Shaffrey C.I., Van Alstyne E.M., Skelly A.C. (2012). Proximal junctional kyphosis as a distinct form of adjacent segment pathology after spinal deformity surgery: A systematic review. Spine.

[9] Hostin R., McCarthy I., O’brien M., Bess S., Line B., Boachie-Adjei O., Burton D., Gupta M., Ames C., Deviren V. (2013). Incidence, mode, and location of acute proximal junctional failures after surgical treatment of adult spinal deformity. Spine.

[10] Maruo K., Ha Y., Inoue S., Samuel S., Okada E., Hu S.S., Deviren V., Burch S., William S.B., Ames C.P. (2013). Predictive factors for proximal junctional kyphosis in long fusions to the sacrum in adult spinal deformity. Spine.

[11] Yagi M., Rahm M., Gaines R., Maziad A., Ross T., Kim H.J., Kebaish K., Boachie-Adjei O. (2014). Characterization and surgical outcomes of proximal junctional failure in surgically treated patients with adult spinal deformity. Spine.

[12] Smith M.W., Annis P., Lawrence B.D., Daubs M.D., Brodke D.S. (2015). Acute proximal junctional failure in patients with preoperative sagittal imbalance. Spine J..

[13] Nguyen N.L.M., Kong C.Y., Hart R.A. (2016). Proximal junctional kyphosis and failure-diagnosis, prevention, and treatment. Curr. Rev. Musculoskelet. Med..

[14] Nicholls F.H.M., Bae J., Theologis A.A., Eksi M.S., Ames C.P., Berven S.H., Burch S., Tay B.K., Deviren V. (2017). Factors associated with the development of and revision for proximal junctional kyphosis in 440 consecutive adult spinal deformity patients. Spine.

[15] Kim J., Phan K., Cheung Z.B., Lee N., Vargas L., Arvind V., Merrill R.K., Gidumal S., Di Capua J., Overley S. (2018). Surgical, radiographic, and patient-related risk factors for proximal junctional kyphosis: A meta-analysis. Glob. Spine J..

[16] Lafage R., Beyer G., Schwab F., Klineberg E., Burton D., Bess S., Kim H.J., Smith J., Ames C., Hostin R. (2019). Risk factor analysis for proximal junctional kyphosis after adult spinal deformity surgery: A new simple scoring system to identify high-risk patients. Glob. Spine J..

[17] Liu F.Y., Wang T., Yang S.D., Wang H., Yang D.L., Ding W.Y. (2016). Incidence and risk factors for proximal junctional kyphosis: A meta-analysis. Eur. Spine J..

[18] Lee S.J., Binkley N., Lubner M.G., Bruce R.J., Ziemlewicz T.J., Pickhardt P.J. (2016). Opportunistic screening for osteoporosis using the sagittal reconstruction from routine abdominal CT for combined assessment of vertebral fractures and density. Osteoporos. Int..

[19] Muraki S., Yamamoto S., Ishibashi H., Horiuchi T., Hosoi T., Orimo H., Nakamura K. (2004). Impact of degenerative spinal diseases on bone mineral density of the lumbar spine in elderly women. Osteoporos. Int..

[20] Lee J.H., Lee J.H., Park J.W., Shin Y.H. (2012). The insertional torque of a pedicle screw has a positive correlation with bone mineral density in posterior lumbar pedicle screw fixation. J. Bone Jt. Surg. Br..

[21] Schreiber J.J., Anderson P.A., Rosas H.G., Buchholz A.L., Au A.G. (2011). Hounsfield units for assessing bone mineral density and strength: A tool for osteoporosis management. J. Bone Jt. Surg. Am..

[22] Pickhardt P.J., Pooler B.D., Lauder T., Rio A.M.D., Bruce R.J., Binkley N. (2013). Opportunistic screening for osteoporosis using abdominal computed tomography scans obtained for other indications. Ann. Intern. Med..

[23] Choi M.K., Kim S.M., Lim J.K. (2016). Diagnostic efficacy of Hounsfield units in spine CT for the assessment of real bone mineral density of degenerative spine: Correlation study between T-scores determined by DEXA scan and Hounsfield units from CT. Acta Neurochir..

[24] Hendrickson N.R., Pickhardt P.J., Rio A.M.D., Rosas H.G., Anderson P.A. (2018). Bone mineral density T-scores derived from CT attenuation numbers (Hounsfield units): Clinical utility and correlation with dual-energy X-ray absorptiometry. Iowa Orthop. J..

[25] Zou D., Li W., Xu F., Du G. (2019). Use of Hounsfield units of S1 body to diagnose osteoporosis in patients with lumbar degenerative diseases. Neurosurg. Focus.

[26] Zou D., Jiang S., Zhou S., Sun Z., Zhong W., Du G., Li W. (2019). Prevalence of osteoporosis in patients undergoing lumbar fusion for lumbar degenerative diseases. A combination of DXA and Hounsfield units. Spine.

[27] Zaidi Q., Danisa O.A., Cheng W. (2019). Measurement techniques and utility of Hounsfield unit values for assessment of bone quality prior to spinal instrumentation: A review of current literature. Spine.

[28] Yao Y.-C., Elysee J., Lafage R., McCarthy M., Louie P.K., Alshabab B.S., Weissmann K., Lafage V., Schwab F., Kim H.J. (2021). Preoperative Hounsfield units at the planned upper instrumented vertebrae may predict proximal junctional kyphosis in adult spinal deformity. Spine.

[29] Duan P.-G., Mummaneni P.V., Rivera J., Guinn J.M.V., Wang M., Xi Z., Li B., Wu H.-H., Ames C.P., Burch S. (2020). The association between lower Hounsfield units of the upper instrumented vertebra and proximal junctional kyphosis in adult spinal deformity surgery with a minimum 2-year follow-up. Neurosurg. Focus.

[30] Genant H.K., Wu C.Y., Kuijk C.V., Nevitt M.C. (1993). Vertebral fracture assessment using a semiquantitative technique. J. Bone Miner. Res..

[31] Uei H., Tokuhashi Y., Maseda M., Nakahashi M., Sawada H., Matsumoto K., Miyakata H. (2018). Exploratory analysis of predictors of revision surgery for proximal junctional kyphosis or additional postoperative vertebral fracture following adult spinal deformity surgery in elderly patients: A retrospective cohort study. J. Orthop. Surg. Res..

[32] Hiyama S., Sasaki D., Katoh H., Sato M., Watanabe M. (2022). Relationship between Hounsfield Units of Upper Instrumented Vertebrae, Proximal Junctional Failure, and Global Alignment and Proportion Score in Female Patients with Adult Spinal Deformity. World Neurosurg..

[33] Zou D., Sun Z., Zhou S., Zhong W., Li W. (2020). Hounsfield units value is a better predictor of pedicle screw loosening than the T-score of DXA in patients with lumbar degenerative diseases. Eur. Spine J..

[34] Buell T.J., Buchholz A.L., Quinn J.C., Bess S., Line B.G., Ames C.P., Schwab F.J., Lafage V., Shaffrey C.I., Smith J.S. (2019). A pilot study on posterior polyethylene tethers to prevent proximal junctional kyphosis after multilevel spinal instrumentation for adult spinal deformity. Oper. Neurosurg..

[35] Theologis A.A., Burch S. (2015). Prevention of acute proximal junctional fractures after long thoracolumbar posterior fusions for adult spinal deformity using 2-level cement augmentation at the upper instrumented vertebra and the vertebra 1 level proximal to the upper instrumented vertebra. Spine.

[36] Ishihara M., Taniguchi S., Adachi T., Kushida T., Paku M., Ando M., Saito T., Kotani Y., Tani Y. (2021). Rod contour and overcorrection are risk factors of proximal junctional kyphosis after adult spinal deformity correction surgery. Eur. Spine J..

[37] Yagi M., Ohne H., Konomi T., Fujiyoshi K., Kaneko S., Komiyama T., Takemitsu M., Yato Y., Machida M., Asazuma T. (2016). Teriparatide improves volumetric bone mineral density and fine bone structure in the UIV+1 vertebra, and reduces bone failure type PJK after surgery for adult spinal deformity. Osteoporos. Int..

[38] Romme E.A., Murchison J.T., Phang K.F., Jansen F.H., Rutten E.P.A., Wouters E.F.M., Smeenk F.W.J.M., Beek E.J.R.V., Macnee W. (2012). Bone attenuation on routine chest CT correlates with bone mineral density on DXA in patients with COPD. J. Bone Miner. Res..

[39] Ton A., Alluri R.K., Kang H.P., Kim A., Hah R.J. (2021). Comparison of Proximal Junctional Failure and Functional Outcomes Across Varying Definitions of Proximal Junctional Kyphosis. World Neurosurg..

